# Death wishes and explicit requests for euthanasia in a palliative care hospital: an analysis of patients files

**DOI:** 10.1186/1472-684X-13-53

**Published:** 2014-11-27

**Authors:** Frédéric Guirimand, Etienne Dubois, Lucy Laporte, Jean-François Richard, Danièle Leboul

**Affiliations:** Pôle Recherche et Enseignement Universitaire SPES (Soins Palliatifs En Société), Maison Médicale Jeanne Garnier, 106 av Emile Zola, 75015 Paris, France

**Keywords:** Palliative care, Palliative care hospital, Euthanasia requests, Wish to die, Suicidal thought

## Abstract

**Background:**

In the current public debate in France about end-of-life and legalization of euthanasia, palliative care is considered as a suitable answer or an alternative or even a supplement to euthanasia. The debate is based on opinion surveys, partly because there is a lack of objective data about the incidence of euthanasia requests (ER) in palliative care settings. The aim of this study was to collect, classify and quantify the expressions of wishes to die (WD), based on computerized files for patients admitted to an 81-bed palliative care hospital (PCH) in Paris during 2010–2011.

**Methods:**

Two researchers analyzed the carers’ notes extracted on the basis of containing the words “wish to die”, “euthanasia” or any expressions relating to death. Notes related to WD and the corresponding patients were then classified in the order: ER, suicidal thought (ST) and other wish to die (OWD). Repeated ER were qualitatively analyzed according to a grid.

**Results:**

We found that 195 of the 2157 patients (9%) expressed a WD: 61 (3%) expressed an ER; 15 (1%) described ST and 119 (6%) expressed an OWD without requiring acting. The WD group was predominantly female, stayed longer in the hospital (median 24 vs. 13 days), and consumed more anxiolytics and antidepressants. None of age, disease or marital status was associated with ER. More women and widows expressed an OWD. Twenty-six ER patients also expressed an OWD and two a ST. Six patients repeated their ER: all had poorly controlled symptoms with repercussions for their mental state.

**Conclusion:**

Our data show the existence of various expressions of WD with a low incidence of ER in a French PCH. The observation of WD including ER is suggestive of good communication between the patients and the care teams. Independent of the changeability of expressions of WD, their very existence should lead to a consideration of the dynamic changes in these WD, and to care staff paying additional attention to the individual, their suffering and the context.

## Background

The possible legalization of euthanasia is currently a question provoking substantial public debate in several countries. In France, the law of April 22^nd^ 2005 concerning the rights of patients at the end of life (the so-called “Léonetti law”) permits the limitation or stopping of treatment, differentiating these situations from euthanasia, which remains prohibited[1]. Public debate is affected by ideological position-taking and political issues, and is essentially fed by emotional reactions to highly publicized individual situations and opinion polls. For example: a poll in October 2012 indicated that 86% of the French population was in favor of the legalization of euthanasia[2]. The first scientific data concerning conditions at the end of life in France have just been published[3, 4]. By contrast, data of this type have been published periodically for almost 20 years in neighboring countries, such as Belgium and the Netherlands, which have since legalized euthanasia if certain circumstances are met[5–10].

A survey of French doctors reported that 1.8% of patients had explicitly requested euthanasia[3]. Although difficult to evaluate, the unbearable nature of suffering at the end of life is one of the principal reasons for such requests, and is indeed an essential criterion for a positive response to such requests in the countries in which this practice has been legalized[11, 12]. Favoring the development of palliative care is often put forward as an alternative in debates on possible changes to laws concerning the end of life, with the risk of reinforcing opposition between palliative care organizations and advocates of euthanasia[13–15]. Despite the development of palliative care in France, most of those calling for the decriminalization of euthanasia or physician-assisted suicide do so in response to intolerable situations they have seen afflicting their relatives or friends at the end of life (report of the French National Ethics Advisory Committee, the CCNE)[14]. Indeed, the impact of palliative care on euthanasia requests (ER) and the stability of such requests remain unclear; in France this issue is still the subject of debate[15–17]. Palliative care hospitals (PCH) manage patients including those in the most complex situations, and have to deal with ER. But, as pointed out by the CCNE report, it would be unrealistic to think that palliative care can deal with all the possible situations of suffering at the end of life and that such care would eliminate all requests to die[14].

A “wish to die” (WD) may be expressed in several ways. Between the wish to not prolong life artificially and an ER, there is a whole range of expressions: the desire not to prolong life, the wish to die “quickly”, the wish to hasten death, the wish to end life, a suicidal thought (ST) and others. The differences in expression and meaning can be subtle such that it may be difficult to distinguish between them, and lead to confusion for care staff and relatives. The aim of this study was to evaluate what carers reported about what the patients said them about their WD. The originality of our work is that it distinguishes between, and quantifies, WD which require an act (ER, or ST) and the other wishes to die (OWD), and considers how they change.

Maison Médicale Jeanne Garnier is a hospital totally dedicated to palliative care, and is recognized for its high level of competence and expertise in end-of-life care. It has 81 beds in six palliative care units (PCU) and admits more than 1100 patients annually. Most (96%) of the patients are in the terminal phase of cancer, with most of the others suffering from progressive neurological diseases. The death rate is 87%, with no significant difference between the six units.

## Methods

There were four steps:

- Extraction of relevant information from medical observations and paramedical notes written by nurses, care assistants, psychomotor therapists and physiotherapists, psychologists and art therapists; all these notes were considered as carers’ notes.
- Analysis by two researchers of context of the extracted carers’ notes
- Classification of each carers’ note into one, and only one, of three groups (ER, ST, OWD) or exclusion.
- Targeted descriptive analysis of inpatient records in cases where the ER was reiterated.

### Definition of the notion of euthanasia and the wish to die

A multidisciplinary working group of doctors, a psychologist and nurses defined the vocabulary before the start of the study. The following definition of euthanasia was adopted: an act deliberately designed to end the life of a person suffering from a serious incurable disease and, at his/her request, put an end to a situation that he/she considers unbearable[14]. This definition excludes requests made to care staff by members of the patient’s family.

The ER group was differentiated from the OWD group; OWD was defined as the expression by the person concerned of wishing to die or to be dead, but without explicitly requesting the assistance of a third party. A wish for the end of life to be short, for time to pass rapidly or to die quickly was classified as OWD; the patient had never explicitly requested an act to accelerate death. Suicidal thoughts (ST group) were classified specifically when the patient used the term “suicide” or talked explicitly about dying at their own hand.

### Data extraction and analysis

All the medical and paramedical records for patients at the PCH are computerized (Osiris software, Corwin). The sociodemographic data for all the patients hospitalized in 2010 and 2011 were collected (SAP Business Objects Web Intelligence software). We then integrated the pathology data, data concerning the characteristics of the stay in the PCH (dates of admission and discharge, duration) and the anxiolytic and antidepressant treatments administered during stays in the PCH. The working group chose to conduct a wide-ranging search, to ensure that the identification of all expressions of WD was as exhaustive as possible. Table 1 indicates the computerized searches carried out on all carers’ notes.

**Table 1:** **Keywords used for the computer extraction of relevant information from carers’ notes**

Searches of all files for one or more terms in French, that could be translated as (not exhaustive)	… this has got to stop…
	… can’t go on …
	…it’s too long…
	…can’t stand it any more…
	… kick the bucket…
	… euthanasia…
	… not live any more…
	… suicide…
All combinations bringing together the two groups of terms	Leave, die, death
	Request, want, quickly, wish, desire

The principal typographic errors and spelling mistakes were taken into account, to broaden the search.

The extracted notes were reread and analyzed in context by two researchers, in a non-random chronological order. The notes provided by social workers were excluded because they often included the word “leave” (*partir*), which (in French) is ambiguous and was mostly used to refer to a return home or transfer to another care structure rather than death.

Each note reporting a WD was classified into one, and only one, of three groups: ER, ST or OWD. Other notes, considered not to reflect a WD, were excluded; for example, all references to a previous ER, conversations with staff about euthanasia, requests from relatives and indications that palliative care was perceived as a type of euthanasia were excluded. The classification of each note into one, and only one, group allowed a hierarchy of items to be established, with explicit ER at the top, followed by ST and then, OWD. If there were several notes for a given patient, the patient was classified according the same hierarchy as the notes (ER, ST and OWD): the different notes for the same patient may reflect changeability in the expression of WD.

### Study of repeated requests

We analyzed the files of all the patients who had formulated an ER and checked for the following elements in all cases of repeated ER during the hospitalization: previous ER in the preadmission file, date and duration of the request during the stay in the PCH, and the person to whom the request was made. The working group established a grid for the qualitative analysis of these files, including the following elements:

- The temporal nature of the request (its duration),
- Disease, symptoms and psychological or spiritual suffering,
- Pharmacological and non-pharmacological treatments,
- The offer and use of sedation,
- Multidisciplinary clinical management of the patients,
- Interactions with the family.

### Statistical analysis

The descriptive statistics are expressed as frequencies and percentages for categorical variables and as means and standard deviations (SD) for age at admission. Durations of stay in the unit are expressed as medians and interquartile ranges (IQR). We used chi^2^ tests, Student’s *t* tests and Mann–Whitney U tests to compare groups, as appropriate for the type of variable. Univariate analyses were carried out to estimate the relative risk (RR) with a 95% confidence interval for belonging to a particular group. The significance threshold was fixed at *p* = 0.05 for two-tailed tests.

### Ethics

The study was approved by a regional ethics committee (Comité de Protection des Personnes CPP Ile de France VIII) under the label of an observational study.

## Results

### Description of the sample

In 2010 and 2011, 2157 patients were admitted to the PCH (Table 2), in which they stayed for a median of 13 days (IQR: 6 to 26 days; Table 3). The analysis included 33,024 medical observations and 195,862 nursing notes, corresponding to a mean of 0.7 medical observations and 4.3 paramedical notes per patient per day (Table 2). The computer search extracted 2080 relevant carers’ notes, corresponding to 917 patients; 1745 notes (84%) were excluded after rereading and contextual analysis.

**Table 2:** **Classification of the patients into the three groups of WD**

	Notes (n = 228,886*)		Patients (n = 2157)	
	Number	%	Number	%
Observations and notes extracted by computer search	2080	1%	917	43%
**Group ER, ST or OWD**	335	0.1%	195	9%
Group ER: Request for euthanasia	100	5%	61	3%
Group ST: Thought of suicide	21	1%	15	1%
Group OWD: Other expression of a Wish to Die	214	10%	119	6%
Withdrawn notes	1745	84%	836	39%

*33,024 and 195,862 notes from medical and paramedical staff, respectively.

Computer extraction of medical observations and carers’ notes. Distribution between the three groups. A note can belong to only one group, with priority to “euthanasia request” (ER), then “suicidal thoughts” (ST) then other expression of a wish to die (OWD). A given patient may be linked to several notes: he/she was classified in the ER group if he/she expressed ER at least once; otherwise, the patient was classified in the ST group if expressing ST at least once; otherwise, the patient was classified in the OWD group.

**Table 3:** **Characteristics of the patients**

		All patients		Wish to die (WD)							
				Total		ER group		ST group		OWD group	
Number of patients		2157		195	9%	61	3%	15	1%	119	6%
Sex	Male	973	45%	68	35%***	25	41%	10	67%	33	28%***
	Female	1184	55%	127	65%***	36	59%	5	33%	86	72%***
Age (mean ± SD)		72 ±14		73 ±14		68 ±15		66±16		77±12	
Marital Status	Married/ cohabiting	1115	52%	91	47%	29	48%	10	67%	52	44%
	Single	312	14%	29	15%	8	13%	2	13%	19	16%
	Widow	471	22%	56	29%	14	23%	3	20%	39	33%**
	Divorced	255	12%	19	10%	10	16%	0	0%	9	8%
Median duration of stay (interquartile)		13 (6–26)		24 (11–41)***		20 (10–35)**		21 (14–50)**		26 (12–44)***	
Antidepressants		776	36%	107	55%***	36	59%***	12	80%***	59	50%***
Anxiolytics		1419	66%	171	88%***	57	93%***	11	73%	103	87%***
Disease											
Cancer		1957	91%	179	92%	56	92%	14	93%	109	92%
Amyotrophic lateral sclerosis		36	2%	4	2%	3	5%	0	0%	1	1%
Other		164	8%	12	6%	2	3%	1	7%	9	8%

Comparison with the general population of all patients.

**p<0.01 versus the rest of the population.

***p<0.001 versus the rest of the population.

### Classification by group

As indicated in Table 2, each of the 335 notes was attributed to one of the three groups; then, the corresponding 195 patients were classified into three groups, according to the defined hierarchy. The ages, marital status and diseases did not differ between these other patients in the PCH (Table 3). However, more of these patients were female, the median duration of stay in the unit was significantly longer, and they were more likely than other patients to be treated with anxiolytics (RR = 3.7; 95% CI: 2.4 – 5.6) and antidepressants (RR = 2.2; 95% CI: 1.7 – 2.8).

There were 100 notes including an explicit ER formulated by 61 patients. These 61 stayed longer in the unit and were more likely than other patients to be treated with anxiolytics (RR = 7.4; 95% CI: 2.7 – 20.4) and antidepressants (RR = 2.6; 95% CI: 1.6 – 4.2). The sex, age, family status and diseases of these patients did not differ from those of other patients. The first request was made to a doctor (51% of cases), a nurse (39% of cases), a psychologist (7% of cases) or a care assistant (3%). This first request was made a median of six days after admission to the hospital (IQR: 2–14). The median interval between the last request and the end of the patient’s stay was seven days (IQR: 3–16 days).

Fifteen patients expressed the idea of suicide (ST group), with no significant difference between this group and other patients for age, marital status, disease or sex despite most being men; however, they were more likely to be given antidepressants (RR = 7.1; 95% CI: 2.0 – 25.2).

The OWD group included the 119 patients who expressed a desire to die: he or she spoke about this desire, wish or determination to die, to “leave” or to pass away, but without requesting the assistance of a third party to accelerate the process. Most of this group was female (RR: 2.1; 95% CI: 1.5 – 3.2) and many were widows (RR: 1.8; 95% CI: 1.2 – 2.5).

### Representation of the changeability of the expression of WD

**Figure 1:**
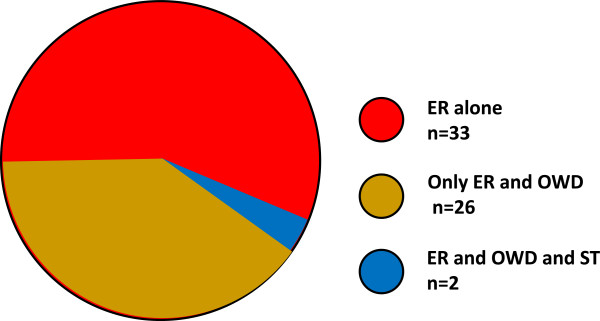
**Patients making an euthanasia request.** The various expressions, at different times, of WD by the patients of the ER group. Red, patients only expressing an ER. Brown, patients expressing both ER and an OWD. Blue, patients expressing ER, ST and OWD.

The notes reporting the ER indicate that 26 of the 61 (43%) patients formulated an OWD at some time; two patients (3%) had also considered killing themselves and, at another time, expressed OWD (see Figure 1).

### Repeated requests for euthanasia

Six of 61 patients reiterated their ER. The characteristics of these patients are summarized in Table 4: four of the six patients were over 83 years old, such that the mean age of this group was nine years older than that of the ER group. All had poorly controlled symptoms, with repercussions for their mental state. For the four oldest patients, the duration of the requests for euthanasia covered between 30 and 100% of their stay in the PCH, with a corresponding number of repeated demands. For the two younger patients (49 and 69 years), the request was repeated only three times over three days. In half these cases, the notion of an ER was recorded in the patient’s preadmission notes.

**Table 4:** **Repeated ER: description of the patients**

Patient	1	2	3	4	5	6
Sex	Female	Male	Male	Male	Female	Female
Age	49	69	85	83	95	84
Disease	Metastatic breast cancer	SLA with ventilation	Metastatic prostate cancer	Metastatic prostate cancer	Metastatic uterine cancer	Pleural cancer
Duration of stay (days)	62	21	33	7	15	45
Request for euthanasia (ER) before admission	Yes	No	No	Yes	No	Yes
Time from admission to first ER (days)	0	14	8	0	2	10
Number of times ER repeated	3	3	5	6	4	6
Time from ER to death (days)	59	5	11	1	5	15
Symptoms	Pain, depression	Dyspnea, respiratory distress	Uncontrolled pain	Diarrhea: complication of irradiation of the small intestine	Major anxiety	Dyspnea, anxiety
Family environment	Separated, one son and one sister	Married, 3 children	Widower, son and stepdaughter, grandchildren	Married	Widow, one son, grandchildren	Widow,son, former daughter-in-law
Multidisciplinary management	Art therapy psychologist psychomotricity physiotherapy hospital visitors	Psychomotricity speech therapy physiotherapy	Psychologist physiotherapy	Refused	Hospital visitors	Psychomotricity volunteers
			social worker			refusal to see a psychologist
Sedation	Not discussed	Intermittent sedation until death	Sedation for a symptom that remained refractory until death	Nocturnal anxiolytics	Anxiolytics	Anxiolytics, refusal of nocturnal sedation.

### Trajectories of the patients repeating the ER

#### Patient 1

On admission, the patient expressed an ER, repeated over three days, subsequently moving towards an expression of the WD. During her long period of hospitalization (62 days) she received reinforced multidisciplinary care and effective antidepressant treatment. Her sadness attenuated and one week before her death she complained that she was sleeping too much.

#### Patient 2

During the first 15 days in the PCH, this patient’s symptoms worsened, with the occurrence of an episode of respiratory distress and an increase in pain: debilitating mycosis, sensations of hunger and thirst, and anguish. Evidence of exhaustion emerged in association with respiratory degradation. The patient became fearful of suffocation, leading to a state of panic. The patient asked very clearly “to die by an injection now”. His demand persisted for three days. Then, consistent with the context of a PCH but with his wife’s opposition, he requested continuous sedation until his death.

#### Patient 3

A combination of several elements were behind this patient’s request for euthanasia nine days after his admission to the unit: a decompensated painful symptom that it was impossible to relieve in a satisfactory manner; a conflict with his daughter that worsened his anger and discomfort; and his determination to not let himself down and to retain his autonomy in decision-making right to the end. The request was repeated over a period of two weeks until in the last week of his life, under the influence of more powerful sedative treatments, he became increasingly sleepy and ceased requesting euthanasia.

#### Patient 4

This 84-year-old patient had been a classical dancer and he found the change in his bodily integrity and major damage to his body image intolerable. From his admission to the unit he requested euthanasia and he continued to repeat this request, showing photographs of himself in his stage costumes, until his death seven days later.

#### Patient 5

The first reference to an ER came from the son of this patient on her admission. The patient herself expressed her wish to die in several different ways. Filled with an intense existential suffering, she requested euthanasia two days after her admission. She repeated this request four times over a period of eight days, but the request was always tinged with ambivalence, as the patient displayed curiosity and pleasure at being alive on a number of occasions. During the five days before her death, her physical weakening and a concomitant decrease in consciousness due to anxiolytic treatment led to a cessation of her requests.

#### Patient 6

The son of this patient transmitted her wish for euthanasia at the time of her admission. For the first week, she was not in pain and suffered little dyspnea. However, the patient’s dyspnea and anxiety then worsened and she began to ask for euthanasia. She formulated an ER after 10 days, which she repeated over a period of three weeks until about two weeks before her death. At the same time, she said that she enjoyed spending time with her children. When she received a positive response to her demand to be transferred to Belgium to have access to euthanasia (this patient was originally from Belgium), her demands ceased and the question of euthanasia was never brought up again. The patient’s symptoms disappeared, and she became comfortable again.

## Discussion

We found that that 9% of the patients hospitalized in our PCH expressed a WD. One in three of these patients expressed an ER, confirming the reality of ER in palliative care settings. We observed changeability in the way the WD was expressed by these inpatients. Persistent ER may be related to uncontrolled symptoms.

### Frequency of ER and OWD in the PCH

Our study confirms the findings of Ferrand who reported requests for hastened death made to French palliative care teams[15]. It documents the occurrence ER at the end of life in a palliative care setting. The low frequency of such requests, 3% of the patients hospitalized in the PCH, is nevertheless higher than the 1.6% reported in 1999 in a French study of 611 patients hospitalized in five PCUs[16]. More recent figures reported by a mobile palliative care team (1500 patients over a period of five years) are closer to ours, with 4.3% of patients requesting euthanasia and 0.6% of patients doing so in a persistent manner[17]; however, this previous study also included requests formulated by relatives. According to a Dutch survey in 2010, there was an explicit ER before 6.7% of all deaths, compared to 4.8% in 2005; this report provided no information about palliative care[9, 18]. A survey of general practitioners in Belgium noted that 27 of 200 non sudden home death patients (13.5%) had at some time formulated an ER; the authors reported that these wishes varied widely between patients, in both their formulation and timing[19]. Other studies are difficult to analyze, as they do not differentiate among WD between ER and OWD[20–22].

We found that 9% of terminally ill patients in our PCH spontaneously expressed a WD, consistent with the frequency reported in a Greek palliative care setting[23]. The value was higher (14 to 17%) when a self-report measure was used to ask all terminally ill patients systematically about their desire for hastened death[20, 24]. These high frequencies contrast with the low frequency of strong WD (2%) reported in a sample of ambulatory cancer patients: therefore, the closeness to death and survival time may make very large contributions to such requests/desires[22]. A WD was clearly associated with depression and hopelessness[20, 24–26]; we found also that antidepressant and anxiolytic consumption by these patients was higher than by other patients. Possibly, this is because the greater needs of these patients are recognized leading to more extensive use of pharmacological solutions.

To our knowledge, the predominance of female patients among those expressing a WD has not been reported before; OWD but not for ER was more frequent among female patients. Women hospitalized in PCHs are more likely to report a lower quality of life[27, 28], and women experience more overall psychological or existential distress than men[29]. This difference between the sexes about WD needs to be confirmed and analyzed, possibly by using a qualitative approach to compare the statements made by male and female patients.

### Requests for euthanasia and palliative care

For more than 20 years, it was generally considered that ER was less likely in palliative care than in other contexts[30–32]; however, this opinion is currently the subject of debate, especially in those countries where euthanasia is legalized[6, 33–35]. In Belgium, euthanasia in response to explicit requests is more frequent in PCU than in other types of care settings[10]. According to Belgian law, palliative care must be provided before euthanasia. One of the characteristics of the practice of palliative care is that there is no unnecessary prolongation of life and that the wishes of the patients are respected, particularly as concerns decisions to stop or limit treatments with no objective other than the prolongation of life[32, 36, 37]. Nevertheless, we show here that there were ER in our PCH. This reflects the good communication between the patients and the care teams. The provision of palliative care might help patients express ER[10]. We found that it takes time (median of 6 days) for this request to emerge and that the patients formulating ER are those staying longer in the PCH. However, according to the EAPC, the situations in which euthanasia or assisted suicide are requested are often complex and therefore the expertise of a palliative care team is required to listen to, understand and support these patients[32]. Consequently, making an ER is one of the reasons for admission into our PCH. The lag before the emergence of the demand may reflect the time required to establish a climate of confidence between the patient and the team in a multidisciplinary environment; indeed, doctors and nurses are approached in 90% of the first requests. This observation raises the issue of the influence of the attitudes of doctors about euthanasia. Kelly showed that demands were more frequent when doctors were in favor of euthanasia[38]. Interestingly, it has been pointed out that the opinion of palliative care and related organizations has, during the last 20 years, moved from opposition to a “studied neutrality”[39]. Note also that the observation of ER may depend on the attentiveness and quality of listening of the caregiver rather than his or her personal convictions or the policy or position of the institution.

### Persistence of inpatients’ requests for euthanasia

We observed that 6 of 61 patients reiterated their ER during hospitalization. Some published studies report that the desire for death and the will to live are highly unstable even over brief periods among cancer patients, especially when they enter the terminal stage[25, 40–42]. According to Ferrand, 34% of ER persist, but the duration of this persistence was not specified. By contrast, patients who express an ER may continue to desire ER after their requests have been refused but they may subsequently keep silent about it[43]. It is important to consider the impact of management on the discontinuity of the expression of the request. Possibly, in a country where euthanasia or physician-assisted-suicide is legal, requests would be more frequent and persistent as the patient is more likely to receive a favorable response from caregivers.

We followed the trajectories of the six patients whose requests for euthanasia were persistent. In four of these patients, decompensation of symptoms (pain, dyspnea, anxiety and depression) was initially of prime importance. These results are consistent with published findings[20, 26, 42, 44]. However, despite the control of symptoms and multidisciplinary management, ERs may persist, probably due to other more complex factors; such factors may reflect the expression of a desire to remain autonomous to the end or the loss of ability to tolerate physical, psychological, social or existential suffering such as a loss of self-esteem[12, 22, 42, 45, 46]. In some cases, the ER only ceases when the patient’s clinical state deteriorates or the patient experiences a decrease in consciousness, possibly a consequence of sedative treatment. In such cases, the change in the patient’s condition prevents him or her from reformulating an ER.

### Wish to die: with or without third party intervention

Our data show the variety of ways the wish not to live longer can be expressed, from an OWD without the intervention of a third party to an explicit ER. Clarification of the difference between an ER and OWD makes it possible to understand the interaction between the two. About two in five patients formulating an explicit ER also express an OWD. This suggests that ER and OWD are in the same psychological register with respect to life: the patient no longer wishes to live. However, the desired mode of death is unclear. The observed changeability of ER should not raise questions about the meaningfulness of these requests, but should lead to consideration of the dynamic changes in these requests, and to care staff paying additional attention to the individual, his or her suffering and the context. Work involving interviews of cancer patients with short life expectancy indicates that wishes for euthanasia or physician-assisted suicide are different from ER: wishes remain hypothetical and are fluctuating and ambivalent[41].

In France, unlike Switzerland, there has not yet been any significant media communication or debate about assisted suicide. This may explain the small number of patients, more male patients than female, thinking of suicide. We found that the patients requesting euthanasia differed from those raising the possibility of suicide, with almost no overlap between these two expressions of WD. This may be linked to differences in patient expectations: euthanasia could be perceived as a “therapeutic act” in the medical field, whereas suicide could be more personal, with a lesser role played by care staff.

### Limitations of this study

Our study is based on the notes in which the care staff recorded WD, and this approach is associated with a risk of underrepresentation. There are three possible causes. First, the resistance to speaking and writing about ER. Although it is routine practice in PCHs to transcribe everything reflecting or describing the complexity of the situation (five notes per patient per day in our institution), care staff confronted with such requests may wish to distance themselves from them, making it difficult for them to transmit this information. Second, carers may feel helpless in response to such demands: they do not lead to any clear action or even decisions. Third, the collection of ER depends upon the sense of WD the carer gave to what he/she heard. Furthermore, only the WD expressed to the staff are included in patient files. Notes were written prospectively but the analyses we report were retrospective. Only a prospective quantitative and qualitative study would provide exhaustive data. Also, our study was conducted in a single hospital. Nevertheless, the hospital includes six PCUs, such that the findings may be representative of palliative care in the Parisian region more generally.

## Conclusion

In the French context of a PCH, we found that WD is expressed by 9% of terminally ill patients, and in various different ways: 6/10 of patients did not request action (OWD); 3/10 formulated an ER and 1/10 thought about suicide. We observed a possible changeability in these modalities of WD expression. Few patients repeated their ER, and ERs were related to uncontrolled symptoms.

The existence of ER in the context of palliative care indicates something of a paradox: the PCH provides an environment attentive to the patient, allowing such requests to emerge; however, it is also an environment intended to ensure that patient care and support are such that demands of this type are unnecessary. The reality is that palliative care teams are confronted by ERs, and this may become more frequent because of possible changes to the law. Our preliminary observations require confirmation in a quantitative and qualitative prospective study taking into account the dynamics of interactions between patients, relatives and care staff.

## Abbreviations

CCNE: Comité Consultatif National d’Ethique (French National Ethics Advisory Committee)
ER: Euthanasia request
OWD: Other wishes to die
PCH: Palliative care hospital
PCU: Palliative care unit
ST: Suicidal thought
WD: Wish to die.
